# Extraction and Concentration of Waste *Pueraria lobata* Stems with Antioxidants and Anti-Melanogenesis Activity as a Novel Skin Whitening Agent for Natural Cosmetic Prototypes

**DOI:** 10.3390/ijms231810352

**Published:** 2022-09-08

**Authors:** Dan Gao, Chong-Woon Cho, Jin-Hyeok Kim, Cheong-Taek Kim, Won-Seok Jeong, Ye Wang, Xiwen Li, Jong-Seong Kang

**Affiliations:** 1 Institute of Chinese Materia Medica, China Academy of Chinese Medical Sciences, Beijing 100700, China; 2 College of Pharmacy, Chungnam National University, Daejeon 34134, Korea; 3 RNS Inc., Daejeon 34134, Korea

**Keywords:** stem of *Pueraria lobata*, anti-melanogenesis, clinical study, cosmetics, bioactive compounds

## Abstract

The root of *Pueraria lobata* (Willd.) is used commercially in different products, including dietary supplements, cosmetics, and teas, but its stem part is rarely used and studied. Therefore, this study evaluated the antioxidant and anti-melanogenesis activities of the bioactive fraction of *P. lobata* stem and investigated whether the activated carbon decolorization technique would have an impact on its activity and chemical composition. We observed that the dichloromethane fraction of *P. lobata* stem (DCM-PLS) has excellent antioxidant and anti-melanin synthesis activity at a concentration of 50 μg/mL. For the investigation of the anti-melanogenesis mechanism, we evaluated the mRNA expression of tyrosinase, which was depressed by the DCM-PLS. Daidzin was identified as the main active ingredient in DCM-PLS by using a high-performance liquid chromatography-diode array detector-hyphenated with tandem mass spectrometry. In addition, the activated carbon decolorization technology has no negative impact on the main components and bioactivity of DCM-PLS. DCM-PLS also did not induce any skin response in the human skin safety test. Collectively, DCM-PLS could be used as a natural type of skin-whitening agent in skin care products.

## 1. Introduction

Oxidative stress has been demonstrated to be one of the principal mechanisms of skin aging and dermatological disease [1]. Ultraviolet radiation from the sun is the most widespread exogenous factor that causes skin aging and damage [2]. Continuous exposure to intense UV radiation not only can trigger the biosynthesis of melanin but also promote the formation of lipid peroxides and reactive oxygen species (ROS) as well as activates several essential enzymes such as elastase, xanthine oxidase and collagenase, which consequently result in changes in connective tissue and lead to a number of skin diseases [3]. The accumulation of free radicals negatively affects the lipids of the extracellular matrix, thus altering the epidermal barrier and contributing to transepidermal water loss, ultimately leading to excessive skin dryness. In addition, lipid peroxidation processes lead to the expression of phospholipases and cyclooxygenases, which then contribute to the synthesis of prostaglandins in the body, which are the main ones responsible for epithelial inflammation. Interestingly, these free radicals can be moderated by a wide variety of beneficial natural plant extracts or compounds known as antioxidants. In this regard, the investigation of the antioxidant and anti-melanogenesis activity of specific materials and their application to alleviate oxidative stress-mediated skin dryness and pigmentation is an effective strategy in the innovation of the cosmetic industry.

In South Korea, China and Japan, a growing number of young women want to prevent non-uniform skin pigmentation and strive for whitening. For example, hydroquinone, kojic acid, azelaic acid, and ascorbic acid have been used as whitening ingredients in cosmetics. Despite the effectiveness of these agents, many of them are deemed to be insecure for utilization in humans. For example, hydroquinone was popularly used in the past to treat hyperpigmentation; however, it has been prohibited by the UK, European, and Japanese governments for use in cosmetics because of its tremendous side effects. Kojic acid is considered to be an active and more secure cosmetic additive, but can also induce side effects, such as dermatitis and erythema, due to high cytotoxicity. Encouragingly, many traditionally used extracts of medicinal plants are considered to be comparatively safe for cosmetic applications [4]. Accordingly, the development of new natural extracts from medicinal plants to develop safer skin whitening agents has drawn increasing attention and support.

*Pueraria lobata* is a perennial plant and is widely used as food and medicine for centuries in Eastern Asian countries [5]. The roots of *Pueraria lobata* have been reported to prevent cardiovascular disease, control diabetes and have strong antioxidant, anti-inflammatory as well as anti-hypertensive activities [6]. However, the vines of *P. lobata* are discarded as waste products and even constitute an environmental issue [7]. Due to its vine weight and extremely rapid growth rate, *P. lobata* is regarded as one of the invasive species and is considered a threat to the ecosystem in the United States and Europe.

Most interestingly, the roots and stems of *P. lobata* have been demonstrated to have a resemblance of phytochemical profiles, and some effective components such as daidzein and daidzin are more abundant in the stems [8]. Previously, we have reported that the ethanol extracts of *P. lobata* stems (PLS) have outstanding anti-melanogenic activity, but unfortunately the crude extract has a deep intrinsic color because it is rich in pigments, which prevents it from being used in the cosmetic industry in South Korea. Therefore, in the present study, we removed the pigments in the dichloromethane fraction of *P. lobata* stem (DCM-PLS) through a series of extraction and purification steps and for the first time used activated charcoal. Further, we also evaluated the effect of DCM-PLS with and without activated charcoal treatment on 3-isobutyl-1-methylxanthine (IBMX)-induced B16/F10 melanoma cells and their antioxidant activity, which will provide a promising strategy for the exploration and utilization of waste *P. lobata* stem resources in cosmetic manufacturing.

## 2. Results

### 2.1. Antioxidant Activity of DCM-PLS

**Figure 1:**
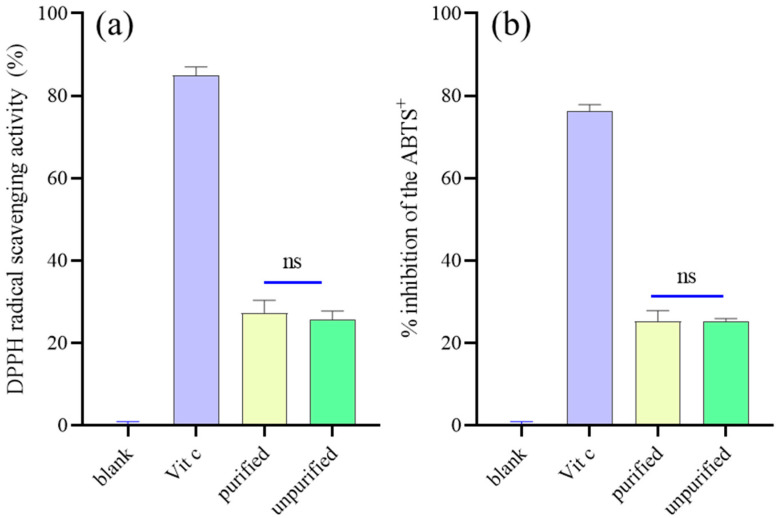
(**a**) DPPH and (**b**) ABTS radical scavenging activity of DCM-PLS purified and unpurified by activated carbon. ns: not significant (*p* > 0.05).

The effect of purification treatment by activated carbon adsorption on the antioxidant activity of DCM-PLS was evaluated by assaying its DPPH and ABTS radical scavenging ability. DPPH radical scavenging capacity of DCM-PLS with and without activated carbon purification was 27.33% and 25.67% (Figure 1a), respectively, showing no significant difference between them (*p* > 0.05). In addition, Figure 1b also shows that treatment with activated carbon does not significantly affect the antioxidant activity of the DCM-PLS. All the results demonstrate that it is feasible to employ activated carbon to remove the pigments from DCM-PLS, and the purified extracts have great potential to be used in the cosmetic industry.

### 2.2. Cell Viability and Anti-Melanogenesis Properties

**Figure 2:**
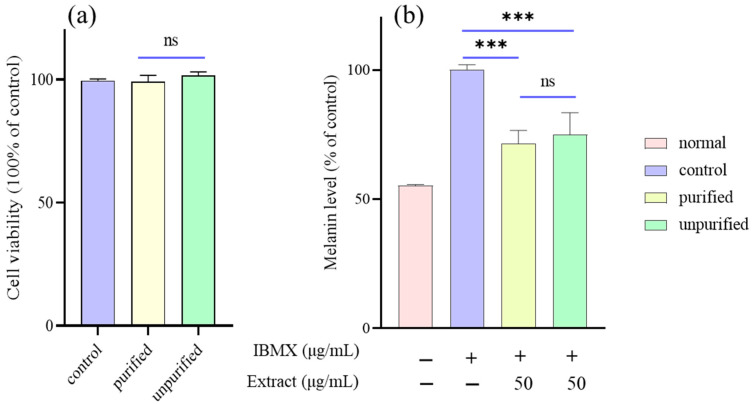
(**a**) Cell viability and (**b**) melanin content in B16/F10 cells treated with DCM-PLS purified and unpurified by activated carbon. *** *p*-value < 0.001 was compared with IBMX induced group. Normal means that the blank group was not stimulated with IBMX. ns: not significant (*p* > 0.05).

The effects of DCM-PLS that had been purified through activated carbon pre- and post-purification on B16/F10 melanoma cell activity were assessed by the EZ-Cytox assay kit. The results have demonstrated that neither the activated carbon treated nor the unactivated DCM-PLS at a concentration of 50 μg/mL exhibit no cytotoxic effects on B16/F10 cell viability (Figure 2a). Thus, a dose (50 μg/mL) without cytotoxicity was selected to determine the effects of DCM-PLS on melanin synthesis. The inhibitory effect on melanogenesis of DCM-PLS with and without activated carbon treatment was similar to those of antioxidant activity evaluation and was shown in Figure 2b. IBMX stimulates the production and release of melanin 2-fold. Interestingly, DCM-PLS with and without activated carbon treatment was found to significantly reduce the melanin level to 71.5 ± 5.1% (*p* < 0.001) and 74.9 ± 8.5% (*p* < 0.001), respectively. Further, there was no statistical difference between the DCM-PLS groups that had been treated with and without activated carbon in terms of inhibition of melanin synthesis.

### 2.3. Visual Observation of Melanin Pigmentations

To better visualize the effect of DCM-PLS on melanin synthesis in the B16/F10 cells, we conducted a visual assessment to show the color changes in the cells. The results suggested that the cells in the IBMX-stimulated group showed a deep black color as compared to the control group, indicating that the synthesis of melanin was successfully induced by IBMX (Figure S1). Interestingly, a noticeable fading of color was observed in cells treated with DCM-PLS purified by activated carbon and IBMX, which suggested that DCM-PLS treated with activated charcoal had extraordinary skin whitening activity.

### 2.4. Inhibition Effects on the Tyrosinase Expression Levels

**Figure 3:**
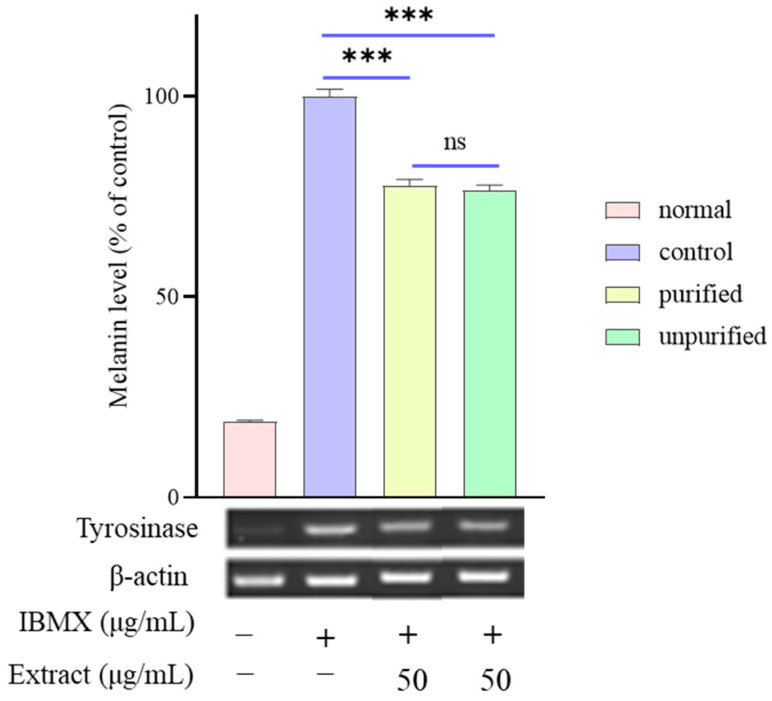
Relative mRNA levels of the melanogenic genes tyrosinase in the B16/F10 cells were quantified using real-time PCR. The β-actin was used as an internal control for equal loading. Data represent the mean ± SD of three independent experiments. *** *p*-value < 0.001 compared with IBMX induced group. ns: not significant (*p* > 0.05).

Melanin biosynthesis requires numerous complicated pathways. Hence, to investigate the potential molecular mechanism of the suppressive effect of DCM-PLS on melanin synthesis, RT-PCR was performed to evaluate the expression levels of tyrosinase that is highly related to melanogenesis in the B16/F10 cells. Figure 3 illustrated that the mRNA expression level of tyrosinase increased 5-fold after being stimulated by IBMX at a concentration of 100 μM compared to the blank group. It is interesting to note that DCM-PLS purified with activated charcoal dramatically down-regulated the tyrosinase gene expression levels to 77.8% compared to cells in the control group (*p* < 0.001). Moreover, both purified and unpurified DCM-PLS by activated carbon had suppressive effects on melanin synthesis, but there was no significant difference between the two groups (*p* > 0.05). These results implied that purified DCM-PLS can be used as an additive in skin whitening cosmetics to ameliorate the problem of melanin deposition in the skin.

### 2.5. Identification and Comparison of Bioactive Components

**Figure 4:**
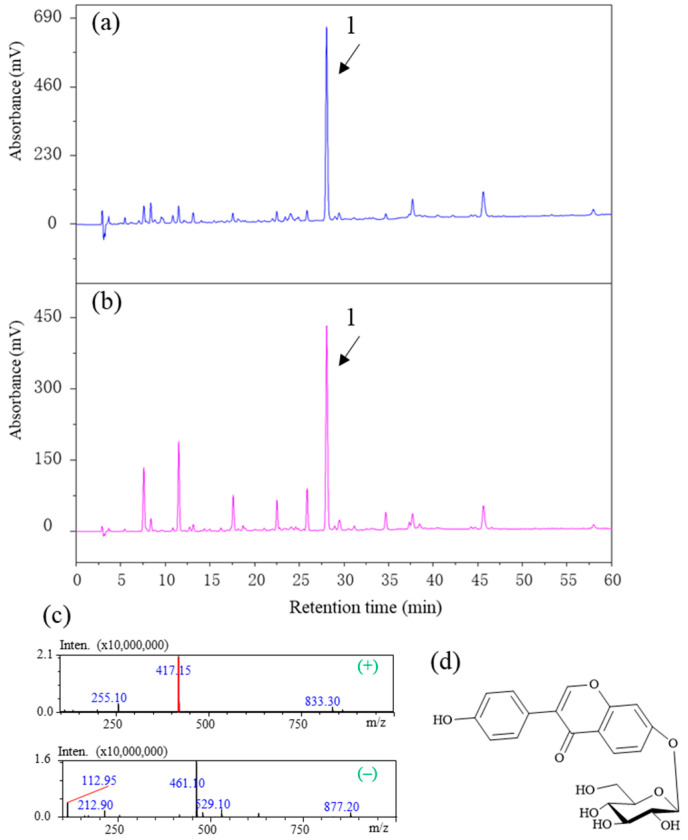
Representative chromatograms of DCM-PLS (**a**) purified and (**b**) unpurified by activated carbon measured at 254 nm. (**c**) MS spectra of peak 1 (daidzin) at positive and negative mode. (**d**) Chemical structure of daidzin. Peak 1: daidzin.

To understand whether the chemical composition of DCM-PLS is affected by activated carbon treatment, an HPLC–diode array detector (DAD)–MS/MS was performed to analyze the changes in the chemical composition of DCM-PLS before and after activated carbon treatment. The HPLC–DAD chromatogram compared the chemical composition changes of DCM-PLS before and after activated carbon treatment and is displayed in Figure 4a,b. The results illustrated that activated carbon can adsorb some chemical components with retention times ranging from 0 to 25 min, and a very distinct peak is present both before and after treatment, which may be the main active component in DCM-PLS contributing to inhibiting the synthesis of melanin. In the MS spectra, peak 1 exhibited pseudomolecular ions [M + H]^+^ and [M + HCOOH–H]^−^ at m/z 417.15 and 461.10 in positive and negative modes, respectively (Figure 4c). The fragmentation of parent ions for peak 1 primarily yields a production by loss of beta-D-glucopyranosyl. On the basis of our previous experimental results and our self-built mass spectrometry database, peak 1 was unambiguously identified as a daidzin, of which structure is presented in Figure 4d.

### 2.6. Skin Irritation Patch Testing

In this study, a total of 32 volunteers participated, and all study subjects faithfully participated in the entire testing process. The mean age of the study subjects was 41.7 ± 6.9 years, with a maximum of 49 years and a minimum of 25 years. For the new whitening cosmetics with DCM-PLS addition, no skin allergic reactions were observed in all subjects after 30 min and 24 h of patch removal. Therefore, the new whitening products made of DCM-PLS were evaluated as hypoallergenic substances in terms of primary irritation to human skin according to the irritation classification criteria.

## 3. Discussion

Efforts to discover new active compounds or extracts with anti-aging, anti-wrinkle, and skin-whiting effects are increasing due to the high demand of society. Thus, investigations of the anti-inflammatory, skin-whitening and antioxidant activities of specific components or natural product extracts are always in progress [9,10,11].

Previous studies have shown that the leaves, stems and roots of *P. lobata* have strong antioxidants in DPPH, ABTS and xanthine oxidase inhibition assays [12]. Phytochemical profiling proved that the stems and roots of *P. lobata* have similar chemical compositions, and the content of daidzin in stems was two times higher than that in roots [8]. The amount of stems that can be acquired from *P. lobata* is two or three times that of roots, and therefore, stems can be employed as a herbal material for manufacturing antioxidants and additives for cosmetics.

Melanogenesis has been widely reported to increase cellular oxidative stress [13]. Several antioxidants, ROS scavengers and inhibitor scavengers may potentially suppress UV-mediated melanin production [14]. Accordingly, antioxidants, ROS scavengers and melanogenesis inhibitors have been applied increasingly in skin-lightening cosmetics to protect against excessive skin pigmentation [15,16]. To elucidate the potential antioxidant activity of DCM-PLS, DPPH and ABTS radical scavenging activity were evaluated. The result demonstrated that DCM-PLS fraction extract exerted satisfactory antioxidant activities in all the analytical studies (Figure 1a,b). In addition, it is very valuable for industrial production that the antioxidant activity of the DCM-PLS does not change after the activated carbon treatment, which suggests that the activated carbon decolorization treatment is safe, reliable and stable. Previously, the essential oil extracted from the leaves of *Artemisia argyi*, vitamin C and vitamin E were reported can reduce the oxidation of pre-existing melanin particles, so these specific materials have been widely used in skin-whiting products [17,18]. Interestingly, in this study, our results demonstrated the antioxidant characteristics of DCM-PLS, indicating that the DCM-PLS purified by activated carbon could act as a skin-whitening ingredient in cosmetics.

The primary consideration in developing a novel agent for ameliorating hyperpigmentation is to assess the safety of the candidate materials using in vitro or in vivo models. Thus, *P. lobata* stem resources were evaluated whether they could meet this criterion. The EZ-Cytox assay kit is widely used to investigate the potential cytotoxicity of specific agents, as these materials increase or inhibit cell viability. The results indicated that the DCM-PLS layer had no cytotoxic effect on B16/F10 melanoma cell viability.

IBMX has been proven to enhance cellular cyclic adenosine monophosphate (cAMP) levels by suppressing phosphodiesterase, a cAMP-degrading enzyme [19]. Furthermore, protein kinase A phosphorylates cAMP-regulatory element binding protein, which binds to the cAMP response element in the promoter region of the microphthalmia-associated transcription factor (MIFT) gene [20]. MITF activation plays a critical role in the triggering of melanogenesis-related genes such as tyrosinase, TRP1 and TRP2. Melanogenesis and mRNA expression of tyrosinase was considerably increased in IBMX-stimulated B16/F10 cells, which were significantly attenuated by DCM-PLS treatment, and were accompanied by diminished melanin production (Figure 2). Moreover, it is encouraging to note that both purified and unpurified DCM-PLS by activated carbon could reduce mRNA expression of tyrosinase in response to induction of IBMX with no statistically significant difference. A cell-based tyrosinase assay will be performed in the future to evaluate the anti-melanogenic activity of DCM-PLS. Similarly to the previous antioxidant results, the activated carbon treatment did not significantly affect the activity of DCM-PLS, which confirms that the activated carbon treatment is stable, reliable and can be used for large-scale industrial production.

In the chemical composition analysis, daidzin was the most abundant component in the DCM-PLS fraction. Importantly, Daidzin has been implicated in the potential inhibition of monophenolase activity of mushrooms with the IC_50_ value of 0.267 ± 0.008 μM [21], which also can inhibit ROS generation, suppress the disruption of zonula occludens-1, and reduce membrane permeability in H_2_O_2_-induced human retinal pigment epithelial cells [22]. Therefore, DCM-PLS rich in daidzin is a good resource for applications in the cosmetic industry.

Our investigation found that DCM-PLS has a satisfactory skin-whitening effect, probably because it is rich in daidzin which has a strong antioxidant capacity and shows no skin primary irritation in humans. We suggest that the DCM-PLS fraction can be used as a possible skin-whiting agent. Unfortunately, DCM use is regulated in some countries for cosmetic applications because it is suspected to be an endocrine disruptor. It could be interesting and important in the future perspective with the claim to search for alternative green solvents to prepare this bioactive extract.

## 4. Materials and Methods

### 4.1. Plant Materials and Regents

The dried stem powders of *P. lobata* were provided by Hanpoong Pharm. Co. Ltd. (Seoul, Korea). The standard of daidzin (purity ≥98%), MS-grade formic acid, 2,2′-azino-bis(3-ethylbenzothiazoline-6-sulfonic acid) diammonium salt (ABTS), 2,2-diphenyl-1-picrylhydrazyl (DPPH) and IBMX were purchased from Sigma-Aldrich (St. Louis, MO, USA). HPLC-grade acetonitrile and water were obtained from Burdick & Jackson (Muskegon, MI, USA).

### 4.2. Preparation of DCM-PLS

**Figure 5:**
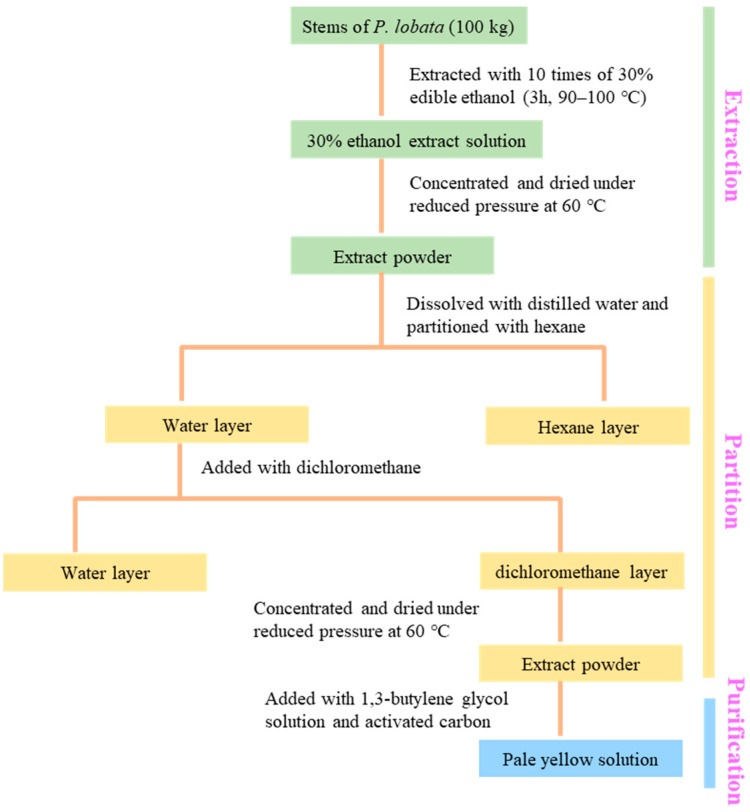
Extraction, partition and purification of fractions from stems of *P. lobata*.

The dried PLS was weighed 100 kg and extracted by using 10 times 30% alcohol of food grade for 3 h at 90 to 100 °C (Figure 5). The extract solution was filtered through a 5 μm filter to remove the fine particle residue and the residue was continued to be extracted twice according to the above conditions, then the filtrate was combined and concentrated under reduced pressure at 60 °C to obtain the dried extract powder. Afterward, the extract powder was added with 10 times distilled water and mixed evenly, then hexane in the same proportion as purified water was added to remove a large number of fat-soluble pigments, then the hexane layer was discarded and the extraction was repeated twice by adding an equal volume of dichloromethane, and all the dichloromethane layers were concentrated under reduced pressure at 60 °C to obtain the dried powder. Finally, dichloromethane layer dried powder (100 g) was added with 500 mL of 1,3-butylene glycol solution as well as activated carbon (50 g) and stirred for 1 h. The obtained mixed pale-yellow solution was passed through a 5 μm filter and finally served as a test solution for future activity evaluation.

### 4.3. Antioxidant Activity

Different prepared DCM-PLS samples were performed to evaluate the scavenging properties of DPPH [23]. The test extracts (50 μg/mL) were added to the DPPH solution at a concentration of 6 × 10^–5^ mol/L and kept at room temperature for 1 h, absorbance of the solution was further measured at 515 nm. ABTS radical cations were produced by reacting ABTS solution (7 mmoL/L) with potassium persulfate and kept in a dark room for 16 h at ambient temperature [24]. Following, extract samples (25 μL) were added to the distilled ABTS solution (250 μL) with ethanol. The absorbance was determined at 734 nm after a 4 min reaction. The percentage of DPPH and ABTS radical scavenging activities were calculated by the following equation: [(1–absorbance of sample/absorbance of negative control) × 100], and ascorbic acid was set up as the positive control.

### 4.4. Cell Culture and Cell Viability Assay

The B16/F10 murine melanoma cells (ATCC^®^ CRL-6475) were obtained from the American Type Culture (ATCC, Manassas, VA, USA). Cells were cultured in Dulbecco’s modified Eagle medium (DMEM, St. Louis, Mo, USA) supplemented with 10% fetal bovine serum and 100 units/mL of penicillin/streptomycin at 37 °C in a humidified atmosphere containing 5% CO_2_. Stock solutions (5 mg/mL) were prepared in dimethyl sulfoxide (DMSO, Sigma, St. Louis, MO, USA) and were then diluted to proper concentrations in the growth medium. Cell viability was evaluated by EZ-Cytox assay kit (Daeil Lab Service Co. Ltd., Seoul, Korea) as described previously [25].

### 4.5. Determination of the Cellular Melanin Contents

Melanin content was determined as described previously with slight modifications [26]. Briefly, the B16/F10 cells (3 × 10^4^) were seeded into a 24-well plate and incubated overnight to allow cells to adhere. The cells were exposed to DCM-PLS with a concentration of 50 μg/mL for 48 h in the absence or presence of 100 μM IBMX. At the end of the treatment, the cells were washed with phosphate buffer saline and lyzed with 800 μL of 1 N NaOH solution containing 10% DMSO for 1 h at 80 °C. The absorbance at 405 nm was measured using a microplate reader (TECAN, Salzburg, Austria). The amount of melanin was expressed as a percentage compared to the control group.

### 4.6. Visual Evaluation of Melanin Pigmentation

The experimental procedure for visual evaluation of melanosis was performed following the method previously recorded [25].

### 4.7. Real-Time Quantitative Polymerase Chain Reaction (RT-PCR) Analysis

The expression levels of a melanogenesis-related gene (tyrosinase) in the B16/F10 cells were measured by RT-PCR according to the previously reported procedure (Figure S2) [27].

### 4.8. Phytochemical Profile

Separation of isoflavonoids from DCM-PLS was performed on a Shimadzu LC-20ADXR high-performance liquid chromatography (HPLC) system (Shimadzu, Kyoto, Japan). This instrument was equipped with an Optimapark C18 column (4.6 mm × 250 mm, 5 μm) from RS Technologies (Seoul, Korea). Acidified water (0.1% formic acid, *v*/*v*) and acetonitrile were used as mobile phases A and B, respectively. A linear-gradient elution was programmed as follows: 0 min, 12% B; 60 min, 43% B, and finally, the initial conditions were kept for 10 min as a re-equilibration step. Parameters for analysis were set using negative and positive ion modes, with spectra acquired over a mass range from m/z 100 to 1000. The optimum values of the electrospray ionization–mass spectrometry (ESI–MS) parameters were: capillary voltage, −3.5 and +3.5 kV; drying gas flow, 15 L/min; desolvation line temperature, 250 °C; nebulizing gas flow rate, 3 L/min, heat block temperature, 400 °C.

### 4.9. Primary Skin Irritation Test

Patch testing was performed to assess the primary skin irritation potentials of the evaluated materials [28]. Thirty-two healthy volunteers participated in this test. The evaluated agents were applied to IQ chambers (Chemotechnique Diagnostics AB, Vellinge, Sweden) and they were then treated in participants for 48 h. Experienced assessors scored participants’ skin reactions at 30 min and 24 h after patch removal based on previously reported grading criteria [28], as follows: 0, no visible reaction; 1, slight spotty or diffuse erythema; 2, moderately uniform erythema; 3, intense erythema with edema; and 4, intense erythema with edema and vesicles. The skin response score (R) was calculated based on the following equation: R = [∑ (score × Numer of Responders)/4 × (maximum score) × N (Total subjects)] × 100. The Human Primary Irritation Index is used to describe the primary skin irritation potential of the test material [29], which was provided in Table S1.

### 4.10. Statistical Analysis

All statistical analyses were carried out using GraphPad Prism (Version 8.02, GraphPad Software Inc., La Jolla, CA, USA). * *p*-value < 0.05, ** *p*-value < 0.01, and *** *p*-value < 0.001 were considered to demonstrate statistically significant differences.

## 5. Conclusions

The utilization of underdeveloped and waste fractions of biorenewable biomass will provide environmental and economic benefits. To investigate the potential of wasted PLS as a skin whitening agent, antioxidant, and anti-melanogenic effects of DCM-PLS on IBMX-induced melanogenesis were performed in B16/F10 melanoma cells. The result suggests that DCM-PLS exhibited significant antioxidant activities in ABTS/DPPH radical scavenging activity and effectively inhibited IBMX-induced melanin production in B16/F10 cells by repressing tyrosinase mRNA expression levels. Activated carbon for decolorization has been proven to be a very stable and reliable process, which does not affect the effectiveness of DCM-PLS. Based on these results activated carbon-treated DCM-PLS could be used as a promising source of anti-melanogenesis agent and as a potential ingredient in pharmaceuticals and cosmeceuticals intended for slowing down skin aging and melanogenesis processes and preventing skin hyperpigmentation disorders.

## Data Availability

Data is contained within the article and Supplementary Materials.

## Supplementary Materials

The following supporting information can be downloaded at: https://www.mdpi.com/article/10.3390/ijms231810352/s1.
