# Supplementary material for: Extraction and Concentration of Waste Pueraria lobata Stems with Antioxidants and Anti-Melanogenesis Activity as a Novel Skin Whitening Agent for Natural Cosmetic Prototypes
Source: Int J Mol Sci. 2022 Sep 8;23(18):10352. doi: 10.3390/ijms231810352 (PMC9499008; doi:10.3390/ijms231810352)
Supplement: Supplementary file 1 [file ijms-23-10352-s001.zip › ijms-1904253-supplementary.pdf]

International Journal of
Molecular Sciences

MDPI

Article

Extraction and concentration of waste *Pueraria lobata* stems with antioxidants and anti-melanogenesis activity as a novel skin whitening agent for natural cosmetic prototypes

Dan Gao [1], Chong-Woon Cho [2], Jin-Hyeok Kim [2], Cheong-Taek Kim [3], Won-Seok Jeong [3], Ye Wang [1], Xiwen Li [1,*] and Jong-Seong Kang [2,*]

[1] Institute of Chinese Materia Medica, China Academy of Chinese Medical Sciences, Beijing 100700, China
[2] College of Pharmacy, Chungnam National University, Daejeon 34134, Korea; chongw113@naver.com
[3] RNS Inc., Daejeon 34134, Korea
* Correspondence: xwli@icmm.ac.cn (X.W.L.); kangjss@cnu.ac.kr (J.S.K.); Tel.: +86-10-8408-4107(X.W.L.); +82-42-821-5928 (J.S.K.)

Table S1. Skin irritation determination criteria.

Mean score	Criteria
0.00–0.87	No/slight irritation
0.00–0.87	Mild irritation
2.43 ~ 3.44	Moderate irritation
>3.45	Severe irritation

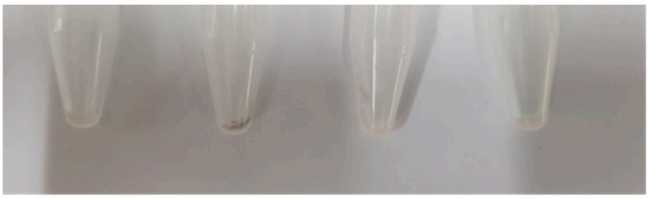

Figure S1. Visual evaluation of each sample in B16F10 cells.

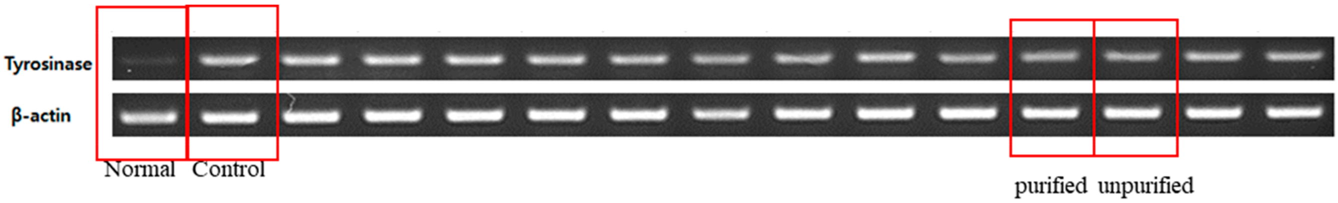

Figure S2. The original gel picture of this experiment.
